# Editorial: Sedentary Behaviors at Work

**DOI:** 10.3389/fpubh.2020.00057

**Published:** 2020-03-10

**Authors:** Frederic Dutheil, Martine Duclos, Yolande Esquirol

**Affiliations:** ^1^Université Clermont Auvergne, CNRS, LaPSCo, Physiological and Psychosocial Stress, University Hospital of Clermont-Ferrand, CHU Clermont-Ferrand, Preventive and Occupational Medicine, WittyFit, Clermont-Ferrand, France; ^2^Université Clermont Auvergne, CRNH, INRA UMR-1019, University Hospital of Clermont-Ferrand, CHU Clermont-Ferrand, Sport Medicine and Functional Explorations, Clermont-Ferrand, France; ^3^Université Paul Sabatier Toulouse 3, INSERM UMR-1027, University Hospital of Toulouse, CHU Toulouse, Occupational and Preventive Medicine, Toulouse, France

**Keywords:** sdentary behaviors, sedentariness, sitting time, intervention, prevention, occupation, job

The aim of this Research Topic on “Sedentary behaviors at work” was to provide a multidisciplinary focus on this area of research. We aimed to give the reader a global overview of sedentary behaviors at work through addressing various aspects. Sedentary behaviors are a leading cause of preventable mortality in developed countries. We mainly have sedentary behaviors at work. Therefore, sedentary behaviors must be considered as an occupational risk, and must be a major concern both for companies/managers and physicians/health researchers (1, 2). However, sedentary behaviors at work were only studied recently over the past decade and many aspects are still poorly studied. This compilation of several relevant original articles and reviews is the result of the contribution of multidisciplinary researchers from several institutes promoting health and prevention. We would like to thank the authors for sharing their exciting work, showing the wide growing interest on this Research Topic, with eminent specialists ranging from medicine and physiology, to psychology, economics, or engineering. Readers may find this editorial a helpful guide to provide an overview of our Research Topic on “Sedentary behaviors at work.”

The health benefit effects of a regular leisure physical activity are well-demonstrated and well-known but the difficulties to meet recommendations defined by World Health Organization remain and thus, physical inactivity affects still a large population of healthy adults. Moreover, the changes, in human activity occurred in the last decades in response to globalization and technology challenges, have transformed occupational and leisure activities toward sedentariness periods at work (prolonged sitting time at office) and also in leisure time (watching TV, video games). Thus, sedentary behaviors become a major concern of modern society and their health detrimental consequences are well-established or strongly suspected on some chronic disease such as cardiovascular disease and cerebrovascular events (Hupin et al.).

## Sedentariness: a Behavior Better and Better Defined

First, sedentariness needs a clear definition (Magnon, Dutheil et al.). Indeed, being physically active does not prevent from having a sedentary behavior. Given the complexity of implicated mechanisms, the distinction between physical inactivity and sedentary lifestyle is important to explain for well-assessing the consequences of sedentary behavior and its interactions with leisure physical activity on health, as debated in two mini-review (Panahi and Tremblay; Thivel et al.).

Given the large part of wake-up time spent at work, people are mainly concerned by sedentariness during their working period.

Therefore, the second question is focused on “how to measure the sedentary ay work?” A systematic review (Boudet et al.) reports several available methods as standardized questionnaires but also more objectively measurements based on visual observations, cardiorespiratory assessment and also more modern technics as global positioning system, smartwatches and smartphones or pressure sensors. Their use in workplace, their advantages and disadvantages have been discussed and interesting recapitulative schemes are provided to clarify the type of available tools.

## Interventional Programs to Reduce Sedentariness: What We do Know?

The last years, preventive interventional programs to fight the sedentariness at work have been emerged in companies to increase the level of physical activity in workplace, in particular among inactive office workers. Several programs are presented and discussed in this topic.

The results obtained after 5- month workplace physical intervention have shown a significant improvement of some anthropometric and fitness measurements among sedentary workers who spent long time/day in front of their computers, findings depending on the level of leisure physical activity at baseline (Genin et al.).

A systematic review was performed to identify the potential effects on cognitive performance when interventions in work environment to reduce sedentariness are implemented. Even if the contrasted results do not allow to conclude firmly to an association between changes in cognitive functioning and the decrease of sedentariness at work, the authors propose several factors to be considered in future researches to improve the homogeneity and the comparison of studies (duration of interventions, daily physical activity, testing time, age, and tools used to measure the sedentariness) (Magnon, Vallet et al.).

To break-up sitting time among desk-based office workers, some new work-stations are proposed but probably insufficiently implemented in companies. These programs question about the facilitators or barriers to implement worksite physical activity and to have a true change in habits. The original qualitative study conducted in thirty employees identified three categories of barriers or facilitators, varying with the stage of change (Planchard et al.). Employees constitute the study- population most often investigated in research studies, however the points of view of employers who allow these potential changes in their companies are also important to consider. From a truck driving population, the perceived value of reducing behavior is explored by interviews. Although, sedentariness is not considered as a priority risk, the potential positive effects of reducing sedentariness programs are reported (Mullane et al.).

A systematic review wants to update our knowledge on a novel concept. Indeed, although it is rarely considered in studies, organizational culture and sedentary behaviors could play a critical role in the success of workplace interventions (Taylor et al.).

Mitigate sitting time by the alternating between stand and sit postures is the aim of most of interventional programs. The ideal solution is currently not found. A United States team explored the preliminary efficacy, preference, and acceptability of two workplace points-of-choice prompt interventions to modify the sedentary behavior among desk-based office workers: a easy and poor cost solution for interesting results! (Larouche et al.).

## And if Sedentariness was a Problem Needing a Change Behavior Very Early in our Life?

Indeed, habitual physical activity level of an adult is also partly determined by the level of physical activity in childhood; examine the possibility to complement the school physical program by promoting physical recreational activity during school recess and measure the effect at long-term (Baquet et al.).

The published articles on this topic help to determine the ways of future researches, notably by contributing to recognize sedentariness at work as an occupational risk and to implement preventive programs in companies. No doubt, that this topic will be developed in up-coming years!

## Author Contributions

FD, MD, and YE contributed to writing this Editorial of the Research Topic on Sedentary Behaviors at Work.

### Conflict of Interest

The authors declare that the research was conducted in the absence of any commercial or financial relationships that could be construed as a potential conflict of interest.
